# Supplementary material for: RISK6, a 6-gene transcriptomic signature of TB disease risk, diagnosis and treatment response
Source: Sci Rep. 2020 May 25;10:8629. doi: 10.1038/s41598-020-65043-8 (PMC7248089; doi:10.1038/s41598-020-65043-8)
Supplement: Supplementary file 1 — Supplementary Figures. [file 41598_2020_65043_MOESM1_ESM.docx]

**RISK6, a 6-gene transcriptomic signature of TB disease risk, diagnosis and treatment response**

Adam Penn-Nicholson^1*^, Stanley Kimbung Mbandi^1*^, Ethan Thompson^2*^, Simon C. Mendelsohn^1*^, Sara Suliman^1,3^, Novel N. Chegou^4^, Stephanus T. Malherbe^4^, Fatoumatta Darboe^1^, Mzwandile Erasmus^1^, Willem A. Hanekom^1^, Nicole Bilek^1^, Michelle Fisher^1^, Stefan H. E. Kaufmann^5,6^, Jill Winter^7^, Melissa Murphy^1^, Robin Wood^8^, Carl Morrow^8^, Ildiko Van Rhijn^3^, Branch Moody^3^, Megan Murray^9^, Bruno B. Andrade^10^, Timothy R. Sterling^11^, Jayne Sutherland^12^, Kogieleum Naidoo^13,14^, Nesri Padayatchi^13,14^, Gerhard Walzl^4^, Mark Hatherill^1^, Daniel Zak^2^, Thomas J. Scriba^1^, and the Adolescent Cohort Study team, GC6-74 Consortium, the SATVI Clinical and Laboratory Team, The ScreenTB and AE-TBC teams, CAPRISA IMPRESS team, RePORT Brazil Consortium and Peruvian Household Contacts Cohort study group.

**Supplementary Figures**

**
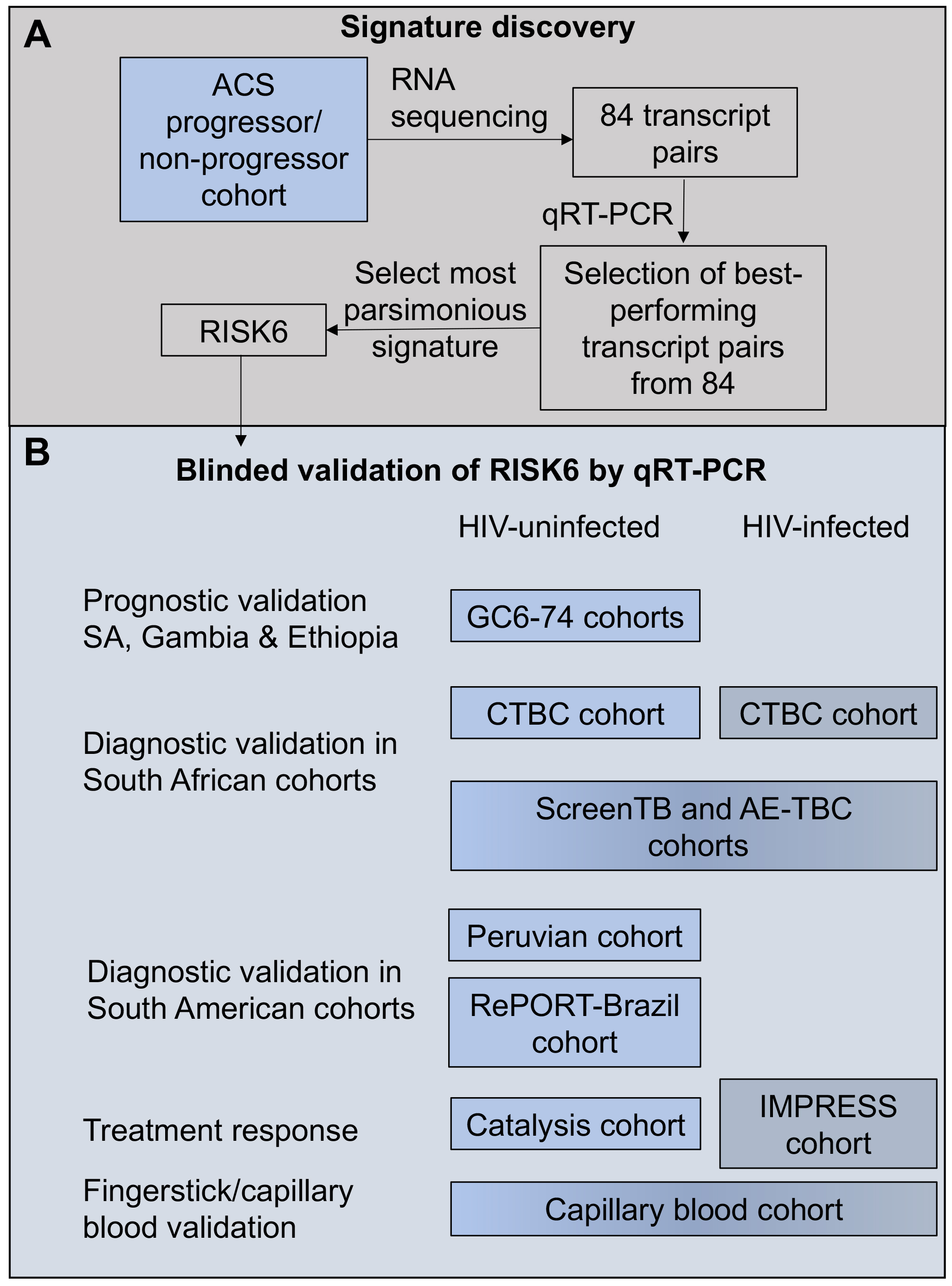
**

**Supplementary Figure 1: Discovery and validation of RISK6**. (A) Schematic depiction of the process to discover the RISK6 signature in TB progressors and non-progressors from the Adolescent Cohort Study, using samples collected within 360 days of TB disease diagnosis ^6^. Differentially expressed exon junctions were identified using RNA-sequencing data and support vector machines of all possible pairs of junctions were trained using the Pair-Ratio approach (see methods). 84 junction pairs that differentiated progressors and non-progressors with the highest sensitivity and specificity were selected and quantified by microfluidic qRT-PCR. Using these PCR data, a small set of junction-pairs was selected to train the most parsimonious signature with excellent performance in the ACS cohort, RISK6. (B) RISK6 was validated by blind application using microfluidic qRT-PCR to samples from seven different cohorts, arranged according to prognostic, diagnostic or treatment response validation and HIV status.


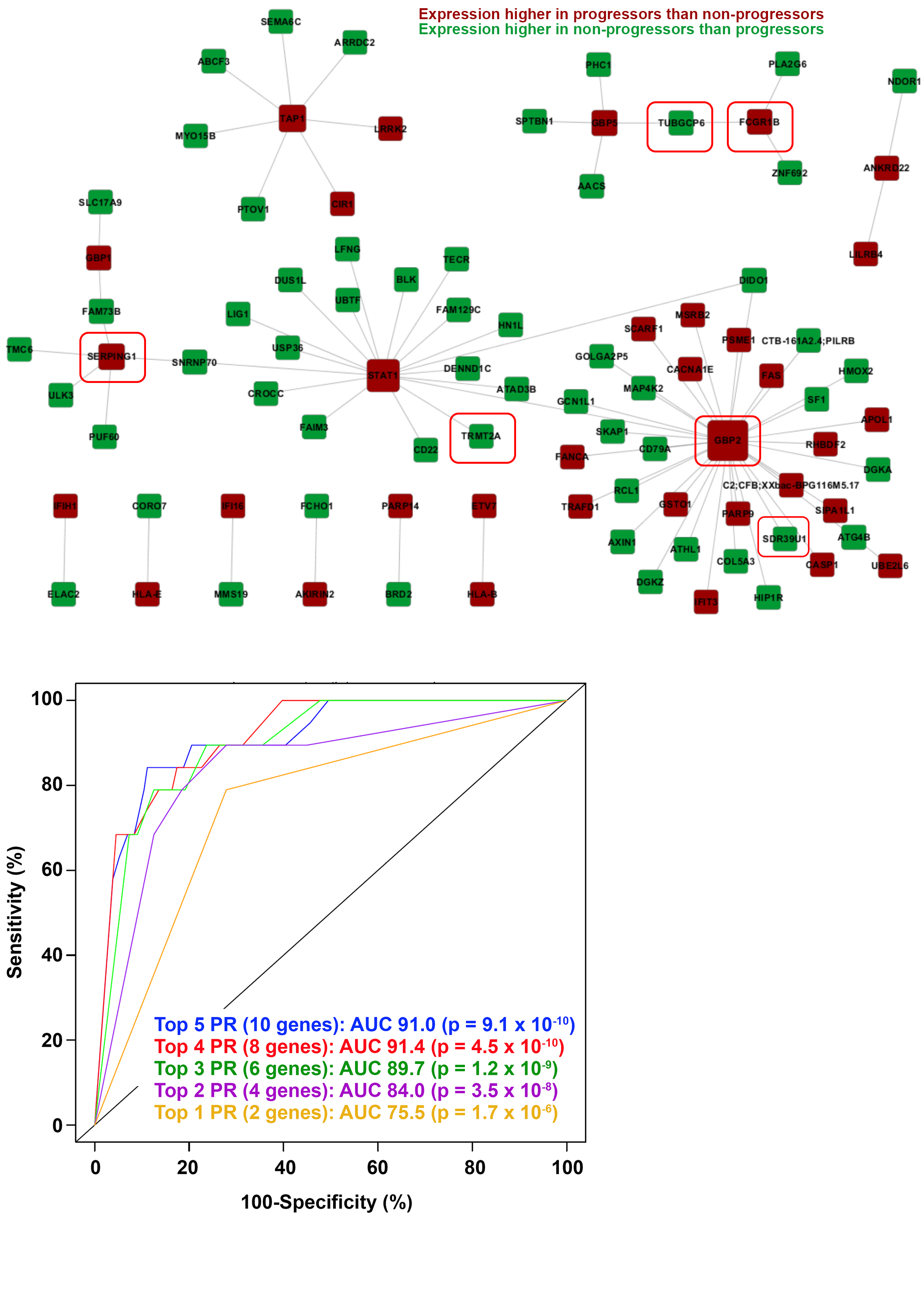


**Supplementary Figure 2**. Network representation of the pairs (depicted by the edges) formed between 84 unique exon junctions (depicted by the nodes) identified from RNA-seq data in the adolescent progressor and non-progressor cohort, by the random subsets approach. This method randomly selects a partition of half the samples with a quarter of the features, to train support vector machines of all possible pairs of junctions using the Pair-Ratio approach. The Pair-Ratio approach pairs transcripts that are regulated in opposite directions in progressors and non-progressors, depicted as red (higher in progressors) and green (higher in non-progressors) nodes. The ROC plot at the bottom depicts the AUCs (and p-values) of 5 parsimonious pair-ratio ensemble signatures comprising between 2 to 10 genes (comprising the top Pair-Ratios, PR), measured by qRT-PCR in samples collected within 6 months of TB diagnosis in ACS progressors versus non-progressors.


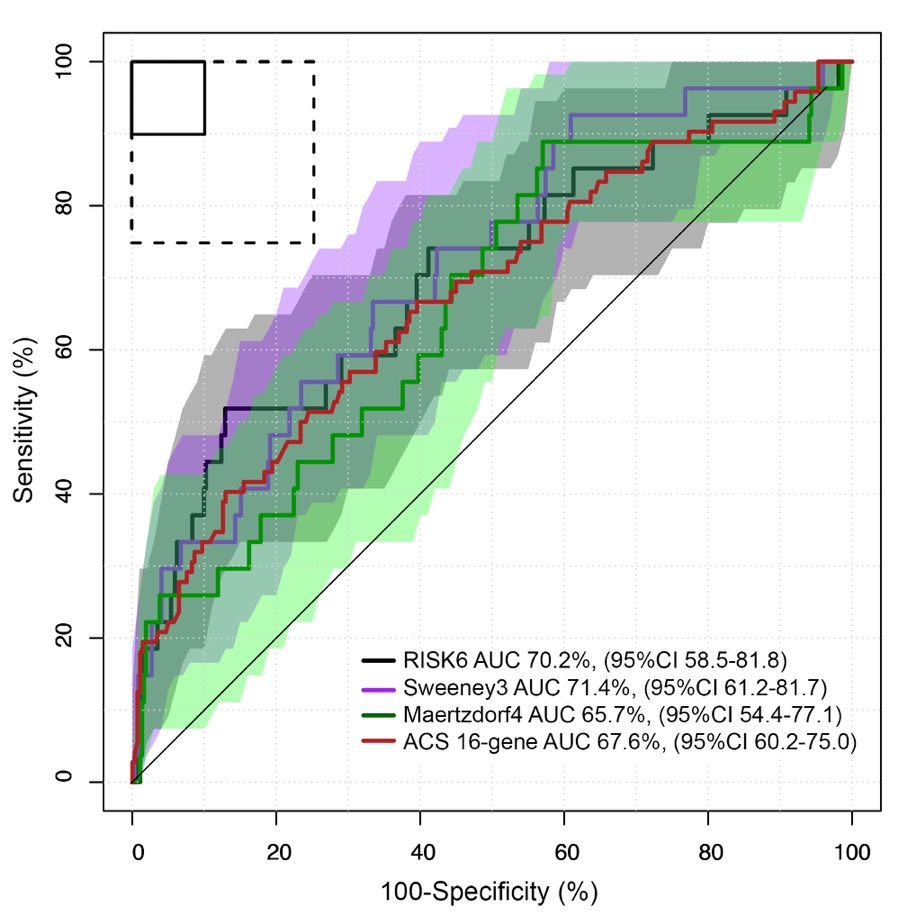


**Supplementary Figure 3**. Area under the ROC curve analysis for transcriptomic signature discrimination between GC6-74 cohort samples from controls and samples from TB progressors collected within 6 months of TB diagnosis. The shading represents 95% confidence intervals. The boxes in the top left corner represent the optimal (solid line) and minimum (dotted line) criteria set out in the target product profile for an incipient TB test. Gene expression was measured by qRT-PCR for all 4 signatures. Methods for measuring the Sweeney3 and Maertzdorf4 signatures are described in Suliman et al., AJRCCM 2018.


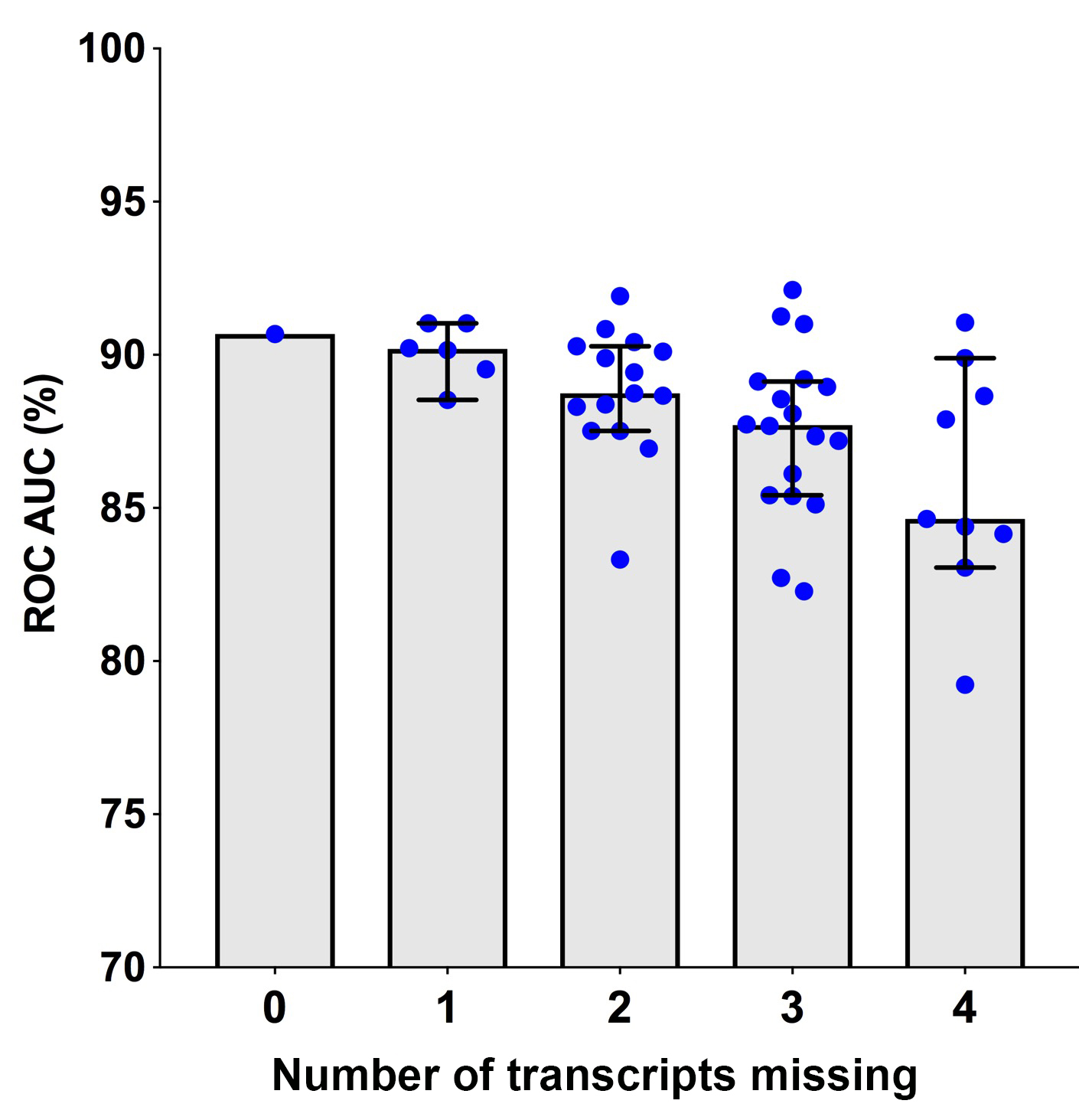


**Supplementary Figure 4**. ROC AUC values for discrimination between TB cases and asymptomatic controls in the Brazilian cohort by the full 6-gene RISK6 signature (nine pairs formed between six transcripts, far left), or after removing 1, 2, 3 or 4, of the transcripts such that every combination of the pairs (represented by individual blue dots) was tested. The grey bar graph represents the mean and the error bar the 95% CI.
